# Distinct Immunoglobulin Fc Glycosylation Patterns Are Associated with Disease Nonprogression and Broadly Neutralizing Antibody Responses in Children with HIV Infection

**DOI:** 10.1128/mSphere.00880-20

**Published:** 2020-12-23

**Authors:** M. Muenchhoff, A. W. Chung, J. Roider, Anne-Sophie Dugast, Simone Richardson, Henrik Kløverpris, Alasdair Leslie, Thumbi Ndung’u, Penny Moore, Galit Alter, Philip J. R. Goulder

**Affiliations:** a Department of Paediatrics, University of Oxford, Oxford, United Kingdom; b HIV Pathogenesis Programme, Doris Duke Medical Research Institute, Nelson R. Mandela School of Medicine, University of KwaZulu-Natal, Durban, South Africa; c Max von Pettenkofer Institute, Virology, National Reference Center for Retroviruses, Faculty of Medicine, LMU München, Munich, Germany; d German Center for Infection Research (DZIF), Partner Site Munich, Munich, Germany; e Department of Immunology and Microbiology, University of Melbourne, The Peter Doherty Institute for Infection and Immunity, Melbourne, VIC, Australia; f Africa Health Research Institute (AHRI), Nelson R. Mandela School of Medicine, University of KwaZulu-Natal, Durban, South Africa; g Department of Infectious Diseases, Ludwig Maximilians University, Munich, Germany; h Rubius Diagnostics, Cambridge, Massachusetts, USA; i Centre for HIV and STI’s, National Institute for Communicable Diseases, Johannesburg, South Africa; j Faculty of Health Sciences, University of the Witwatersrand, Johannesburg, Gauteng, South Africa; k Department of Immunology and Microbiology, University of Copenhagen, Copenhagen, Denmark; l Department of Infection and Immunity, University College London, London, United Kingdom; m Max Planck Institute for Infection Biology, Berlin, Germany; n The Ragon Institute of Massachusetts General Hospital, Massachusetts Institute of Technology, and Harvard University, Cambridge, Massachusetts, USA; National Institute of Allergy and Infectious Diseases

**Keywords:** Fc effector functions, Fc glycosylation, HIV, broadly neutralizing antibodies (bnAbs), nonneutralizing antibodies, pediatric, vaccine

## Abstract

A prophylactic HIV vaccine would ideally induce protective immunity prior to sexual debut. Children develop broadly neutralizing antibody (bnAb) responses faster and at higher frequencies than adults, but little is known about the underlying mechanisms or the potential role of Fc-mediated effector functions in disease progression. We therefore performed systems immunology, with immunoglobulin profiling, on HIV-infected children with progressive and nonprogressive disease. Pediatric nonprogressors (PNPs) showed distinct immunoglobulin profiles with an increased ability to elicit potent Fc-mediated natural killer (NK)-cell effector functions. In contrast to previous reports in adults, both groups of children showed high levels of gp120-specific IgG Fc glycan sialylation compared to bulk IgG. Importantly, higher levels of Fc glycan sialylation were associated with increased bnAb breadth, providing the first evidence that Fc sialylation may drive affinity maturation of HIV-specific antibodies in children, a mechanism that could be exploited for vaccination strategies.

**IMPORTANCE** To protect future generations against HIV, a vaccine will need to induce immunity by the time of sexual debut and hence requires immunization during childhood. Current strategies for a prophylactic HIV vaccine include the induction of a broadly neutralizing antibody response and the recruitment of potent effector functions of immune cells via the constant antibody Fc region. In this study, we show that nonprogressing HIV-infected children mounted antibody responses against HIV that were able to mediate potent Fc effector functions, which may contribute to the control of HIV replication. Children who had specific glycan structures on the Fc portion of antibodies against HIV were able to neutralize a broader range of HIV variants, providing evidence of a potential role of Fc glycovariation in the development of bnAbs against HIV. These findings complement our knowledge of the distinct immune landscape in early life that could be exploited in the development of vaccine strategies.

## Abstract (precis)

To protect future generations against HIV, a vaccine will need to induce immunity by the time of sexual debut and hence requires immunization during childhood. Current strategies for a prophylactic HIV vaccine include the induction of a broadly neutralizing antibody response and the recruitment of potent effector functions of immune cells via the constant antibody Fc region.

## INTRODUCTION

HIV infection in early life typically results in more rapid disease progression than in adults (1). However, there is a subgroup of vertically HIV-infected pediatric nonprogressors (PNPs) who maintain normal-for-age CD4 counts despite ongoing viral replication at high rates in the absence of antiretroviral therapy (ART) (2). This “nonprogressing” phenotype in children is characterized by low levels of immune activation that are maintained by active tolerogenic mechanisms, including increased regulatory T-cell activity and homeostatic signaling (3).

The unique pediatric immune landscape in HIV infection is highlighted by the observation that infected children develop broadly neutralizing antibody (bnAb) responses faster, at higher rates, and with substantially more potency than adults (2, 4). This is also true for vaccine-induced immunity, as a direct comparison between infant and adult responses to the same gp120 vaccine showed that HIV-uninfected children made higher-magnitude antibody responses (5). Efforts to investigate the immunological mechanism that underlies this phenomenon indicate that T follicular helper (Tfh) cells, a subgroup of CD4 T cells specialized to provide help to B cells in antibody affinity maturation and class switching, may play an important role (6). Although they primarily exert their function within germinal centers of secondary lymphoid tissue, various antigen-experienced subsets of CD4 T cells expressing the follicular homing marker CXCR5 can be identified in circulation (7). These cells can provide B-cell help, and the PD-1^+^ CXCR3^−^ CXCR5^+^ subset, which has been associated with HIV neutralization breadth in both adults (7) and children (8), is present at significantly higher frequencies in blood in the latter group.

Besides their neutralizing activity, antibodies elicit immune effector functions by engaging complement factors or Fc receptors expressed on innate immune cells such as natural killer (NK) cells and macrophages, triggering a range of functions, including antibody-dependent cellular cytotoxicity (ADCC) and phagocytosis (ADCP) (9). With regard to HIV, it has been shown that nonneutralizing IgG antibodies that mediate ADCC and ADCP against Env correlate with protection from infection in the RV144 trial (10, 11). Fc-mediated antibody responses have repeatedly been associated with control of viremia and/or delayed disease progression in adults (12–14), but studies of HIV-infected children remain scarce. Previous studies examined the potential effects of ADCC activity by passively acquired maternal IgG on vertical HIV transmission risk and clinical outcome during infancy but not of *de novo* responses during childhood (15–17). Fc-mediated functions, including ADCC, ADCP, as well as the activation of the complement system, are modulated by biophysical aspects of an antibody: isotype/subclass and glycosylation patterns (reviewed in reference 18). All IgGs have an N-glycan attached at N-297 in the CH_2_ region, which can influence antibody stability and Fc receptor and complement binding (9). While the underlying immunological mechanisms controlling antibody glycosylation are poorly understood, variations of bulk glycosylation patterns under distinct physiological conditions like age, sex, and pregnancy have been observed (19). Under pathological conditions, agalactosylated and asialylated carbohydrate forms of antigen-specific IgG have been associated with chronic inflammatory autoimmune diseases (20) and chronic HIV infection (21). The role of Fc-mediated immunity and, in particular, its regulation via glycosylation in children with HIV infection remains largely undefined. Children represent an important target population for a prophylactic HIV vaccine due to their superior ability to both elicit bnAb responses as well as achieve protective immunity against HIV acquisition before sexual debut with puberty. It is therefore crucial to better understand humoral immunity, including the generation of both broadly neutralizing and Fc-effector-mediated effector functions and their regulation in this age group.

In order to identify immunological signatures that are associated with disease nonprogression and the development of broadly neutralizing antibody responses, here, we explore HIV-specific IgG function and glycosylation together with cellular immunity in vertically HIV-infected children with progressive and nonprogressive disease phenotypes.

## RESULTS

### Increased p24- and decreased gp41-specific IgG titers in PNPs.

**FIG 1:**
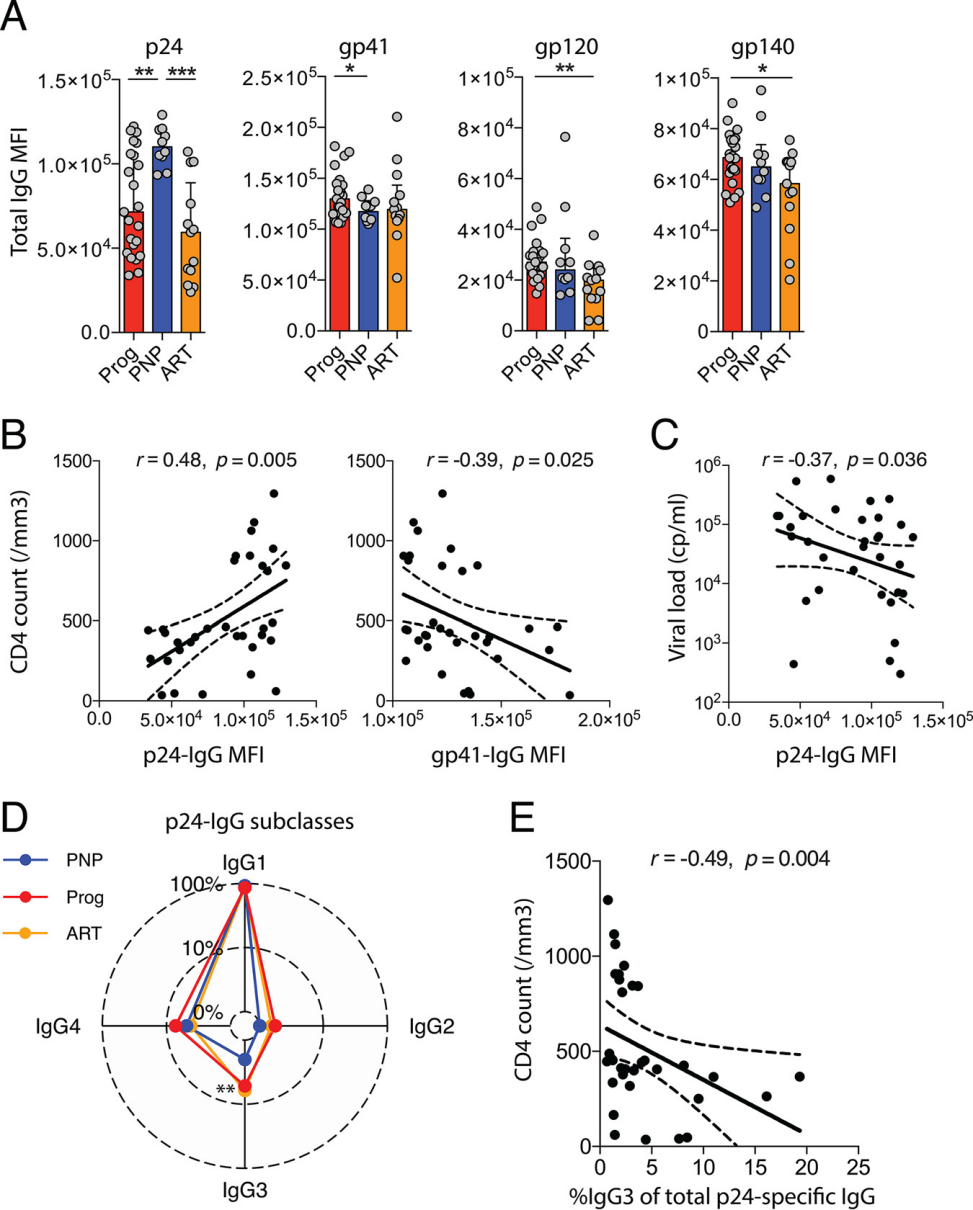
Levels of HIV-specific antibody titers differentiate HIV-infected progressor children and pediatric nonprogressors. (A) Titers (median fluorescence intensities [MFI]) of p24-, gp41-, gp120-, and gp140-specific IgG for HIV-infected progressor children (Prog) (red) (*n* = 23), pediatric nonprogressors (PNP) (blue) (*n* = 10), and ART-treated children (ART) (orange) (*n* = 13). Medians and interquartile ranges are shown as horizontal bars and error bars. Statistical comparisons between groups are based on Mann-Whitney U tests (*, *P* < 0.05; **, *P* < 0.01; ***, *P* < 0.001; ****, *P* < 0.0001). (B and C) Spearman rank correlations between p24- or gp41-specific IgG and CD4 counts (B) or viral loads (C) of progressor and nonprogressor children with trend lines and 95% confidence intervals (dotted lines). (D) Radar plot describing the distributions of different IgG subclasses relative to total p24-specific IgG in sera from HIV-infected progressor children (Prog) (*n* = 23), pediatric nonprogressors (PNP) (*n* = 10), and ART-treated children (ART) (*n* = 13). Statistical comparisons between groups are based on Mann-Whitney U tests (**, *P* < 0.01). (E) Spearman rank correlation between the ratio of p24-specific IgG3 to the total p24-specific IgG response and CD4 counts with trend lines and 95% confidence intervals (dotted lines).

To determine if disease nonprogression in vertical HIV infection is associated with distinct HIV-specific IgG levels, we profiled IgG responses to recombinant HIV-1 consensus C p24, gp41, gp120, and gp140 antigens in HIV-infected pediatric nonprogressors (PNPs) in comparison to progressors or ART-treated children. Consistent with our previous work (2), we defined PNPs as ART-naive, HIV-infected children or adolescents above 5 years of age with CD4 T-cell counts of >750 cells/mm^3^ who have not met clinical or CD4 criteria in the past to initiate ART. Progressor children were defined as ART-naive children above 5 years of age with <500 CD4 T cells/mm^3^, and ART-treated children (ART) were defined as children currently receiving ART who have undetectable viral loads (Table 1). PNPs showed higher levels of p24-specific IgG than progressors and ART-treated children (*P* = 0.005 and *P* = 0.0002, respectively) (Fig. 1A) but lower titers against gp41 than progressors (*P* = 0.046). Responses against gp120 and gp140 were lower in ART-treated children than in progressors (*P* = 0.002 and *P* = 0.02). The significance threshold after Bonferroni correction for the number of these comparisons (12 comparisons, *P* = 0.0042) is met only for the comparisons of p24 IgG levels between PNPs and ART-treated children and gp120 levels between progressors and ART-treated children.

**TABLE 1:** Clinical characteristics of study participants^*a*^

Group	Inclusion criteria	No. of participants	Median age (yrs) (IQR)	No. of females/total no. of participants	Median no. of CD4 cells (IQR)	Mean % CD4 cells (IQR)	VL (cp/ml)	Median time on ART (yrs) (IQR)
Prog	Vertically HIV infected, >5 yrs of age, ART naive, CD4 count of <500 cells/mm^3^	23	13.1 (9.1–16.8)	11/23	378 (251–442)	17 (9–21)	52,000 (7,800–140,000)	None
PNP	Vertically HIV infected, >5 yrs of age, ART naive, CD4 count of >750 cells/mm^3^	10	9.5 (7.5–12.1)	5/10	907 (845–1,076)	34.5 (31–43)	50,500 (875–72,750)	None
ART treated	Vertically HIV infected, >5 yrs of age, ART treated, VL of <20 cp/ml	13	10.1 (8.5–11.7)	6/13	741 (500–939)	30 (22–40)	All <20	3.9 (0.8–5.4)
HIV^−^	HIV negative, >5 yrs of age	10	11.5 (8.6–14.5)	5/10	ND	ND	ND	None

a Median values and interquartile ranges are shown for each clinical parameter. Prog, progressors; HIV^−^, HIV negative; PNP, pediatric nonprogressor; VL, viral load; cp/ml, HIV RNA copies per milliliter of plasma; ND, not determined.

Levels of p24 IgG were positively correlated with CD4 counts (*r* = 0.48; *P* = 0.005), while gp41 IgG1 levels showed an inverse correlation (*r* = −0.4; *P* = 0.02) (Fig. 1B). Only the association between CD4 counts and p24 IgG levels met the significance threshold after Bonferroni adjustment (4 comparisons, *P* = 0.0125). Of note, p24 IgG levels were also negatively correlated with viral loads (*r* = −0.37; *P* = 0.036) (Fig. 1C). As expected, p24-specific responses consisted mainly of IgG1, but we observed lower proportions of p24-specific IgG3 responses in PNPs than in progressors and ART-treated children (*P* = 0.12 and *P* = 0.0015) (Fig. 1D). Correspondingly, the ratio of IgG3 to the total p24-specific IgG response is inversely correlated with the CD4 count (*r* = −0.49; *P* = 0.004) (Fig. 1E), meeting the Bonferroni-adjusted significance threshold (4 comparisons, *P* = 0.0125).

### Limited HIV-specific CD4 T-cell activity in PNPs.

**FIG 2:**
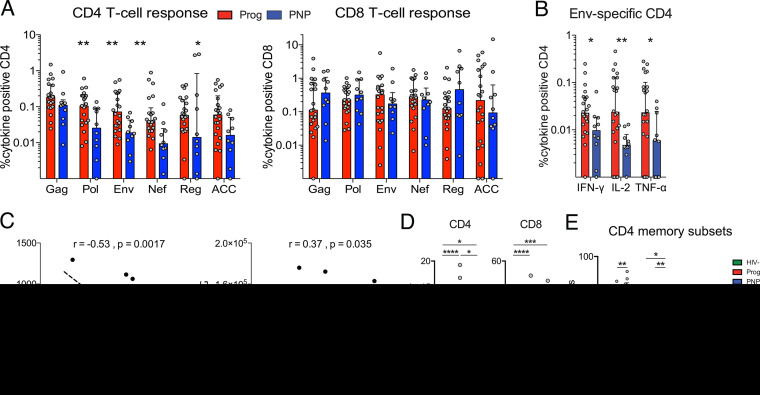
Increased T-cell responses and activation in progressor children. (A) Frequencies of CD4 (left) and CD8 (right) T cells responding with IFN-γ, interleukin-2 (IL-2), and/or TNF-α after stimulation with HIV-1 subtype C Gag, Pol, Env, Nef, regulatory (Reg) (Tat and Rev), or accessory (ACC) (Vpr, Vpu, and Vif) peptide pools. Medians are shown by bar graphs, and interquartile ranges are shown by error bars. Statistical comparisons between groups are based on Mann-Whitney U tests (*, *P* < 0.05; **, *P* < 0.01). (B) Individual cytokine responses for CD4 T cells after stimulation with pooled HIV Env peptides. (C) Spearman rank correlations between frequencies of Env-specific CD4 T cells and CD4 counts or gp41-specific IgG titers with trend lines and 95% confidence intervals (dotted lines). (D) Frequencies of activated (CD38^+^ HLADR^+^) cells gated on live CD3^+^ CD4^+^ or CD8^+^ T cells from HIV-infected progressor (Prog) (*n* = 23) and nonprogressor (PNP) (*n* = 10) children compared to HIV-uninfected children (HIV^−^) (*n* = 10). Median values are indicated by bar graphs, and interquartile ranges are indicated by error bars. Statistical comparisons between groups are based on Mann-Whitney U tests (*, *P* < 0.05; **, *P* < 0.01; ***, *P* < 0.001; ****, *P* < 0.0001). (E) Frequencies of naive (CD45RA^+^ CCR7^+^), effector memory (Tem) (CD45RA^−^ CCR7^−^), central memory (Tcm) (CD45RA^−^ CCR7^+^), and Temra (CD45RA^+^ CCR7^−^) CD4 T cells (gated on live CD3^+^ cells) expressed as medians with interquartile ranges. Statistical comparisons between groups are based on Mann-Whitney U tests (*, *P* < 0.05; **, *P* < 0.01).

To address if these differences in IgG levels were correlated with HIV-specific CD4 T-cell activity, we examined T-cell responses upon stimulation with peptide pools covering all HIV-1 consensus subtype C proteins using intracellular cytokine staining against interferon gamma (IFN-γ), tumor necrosis factor alpha (TNF-α), and interleukin-2 (IL-2). We observed overall higher frequencies of HIV-specific CD4 T cells in progressors than in PNPs, with statistically significant differences for Pol, Env, Nef, and accessory (ACC) peptide pool responses (*P* = 0.008, *P* = 0.004, *P* = 0.002, and *P* = 0.03, respectively) (Fig. 2A). The comparisons between Pol and Env CD4 responses met the significance threshold after Bonferroni adjustment for multiple comparisons (6 comparisons, *P* = 0.009). However, no significant differences were observed for CD8 T-cell responses. A higher frequency of Env-responding CD4 T cells in progressors was observed for all three Th1 effector cytokines tested (IFN-γ, *P* = 0.02; IL-2, *P* = 0.0068; TNF-α, *P* = 0.04) (Fig. 2B). In addition, there is an inverse correlation between the frequency of Env-specific CD4 T cells and CD4 counts (*r* = −0.53; *P* = 0.0017) and a positive correlation with gp41 titers (*r* = 0.37; *P* = 0.035) (Fig. 2C). Individual cytokine responses for the other HIV peptide stimulations are presented in Fig. S1 in the supplemental material, showing the same pattern with lower frequencies of HIV-specific CD4 responses in PNPs.

10.1128/mSphere.00880-20.1 FIG S1 HIV-specific T-cell responses. Frequencies of CD4 (A) and CD8 (B) T cells responding with IFN-γ, interleukin-2 (IL-2), and TNF-α after stimulation with HIV-1 subtype C Gag, Pol, Env, Nef, regulatory (“Reg”) (Tat and Rev), or accessory (“ACC”) (Vpr, Vpu, and Vif) peptide pools. Progressor children (Prog) are shown in red, and nonprogressor children (PNP) are shown in blue. Medians are shown by bar graphs, and interquartile ranges are shown by error bars. Statistical comparisons between groups are based on Mann-Whitney U tests (*, *P* < 0.05; **, *P* < 0.01). Download FIG S1, PDF file, 0.1 MB. Copyright © 2020 Muenchhoff et al. 2020 Muenchhoff et al. This content is distributed under the terms of the Creative Commons Attribution 4.0 International license.

Chronic immune activation is a cardinal feature of progressive HIV infection, whereas PNPs are characterized by low levels of immune activation (2). As described above, progressors showed higher levels of CD4 and CD8 T-cell activation than did HIV-uninfected children and PNPs, while PNPs maintained lower levels of T-cell activation and a less terminally differentiated CD4 T-cell compartment (Fig. 2D and E) (2).

### IgG glycosylation signatures correspond to increased immune activation in progressors.

**FIG 3:**
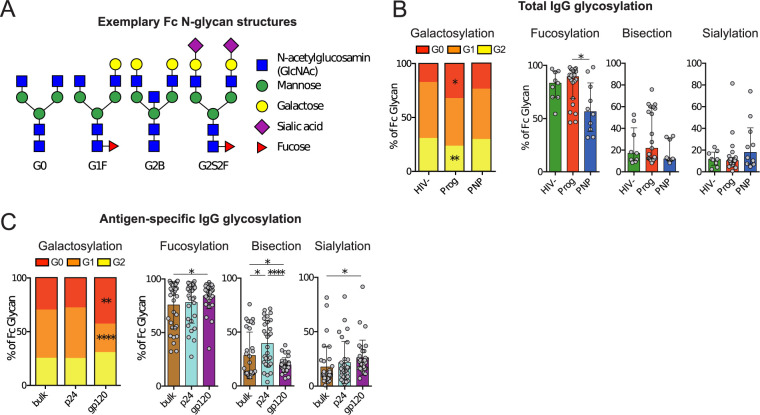
Fc glycosylation is altered by progressive HIV infection. (A) Examples of different IgG Fc N-glycan structures presented using standard symbolic representations. (B) Fc glycosylation of bulk IgG. Galactosylation is shown in stacked bars as the normalized mean for each category of agalactosylated (G0), monogalactosylated (G1), and digalactosylated (G2) glycans. *P* values are indicated for the comparison between progressors and HIV-uninfected children using Mann-Whitney U tests (*, *P* < 0.05; **, *P* < 0.01). Proportions of sialylated, fucosylated, and bisected glycan structures are expressed as medians with interquartile ranges. (C) Fc glycosylation for bulk IgG (brown) in comparison with p24 (cyan)- and gp120 (purple)-specific IgG. Pooled data from all HIV-infected children are presented.

Considering the changes in IgG glycosylation patterns reported for patients with chronic inflammation due to autoimmune diseases (20, 22, 23) and other chronic inflammatory diseases like tuberculosis (TB) (24) or HIV infection (21), we assessed if there are differences in IgG glycosylation between progressors and PNPs and studied IgG glycan structures by a modification of capillary electrophoresis (25). Examples of observed natural glycoforms are shown using standard symbolic representations and nomenclature in Fig. 3A. As expected, given their immune activation status, in progressors, agalactosylated (G0) IgG glycans were expanded (*P* = 0.05), and digalactosylated (G2) glycans were decreased (*P* = 0.0074) compared to HIV-uninfected children (Fig. 3B). The Bonferroni-adjusted significance threshold was not met (9 comparisons, *P* = 0.0045). However, PNPs showed no significant perturbations in bulk IgG galactosylation compared to HIV-uninfected children, highlighting that low immune activation and inflammation are global immunological features of nonprogressive pediatric HIV infection also present in the B-cell compartment.

To study IgG Fc glycovariation at the antigen-specific level, p24- and gp120-specific antibodies were affinity purified before glycosylation analysis. While we observed no differences in antigen-specific glycosylation between PNPs and progressors (Fig. S2), the IgG glycosylation patterns differed for antigen specificity. Compared to bulk IgG, gp120-specific IgGs showed high fractions of agalactosylated (G0) structures at the expense of G1, while no differences were observed for p24, indicating differential regulation of glycan structures against different antigens of the same pathogen (Fig. 3C). These differences met the significance threshold adjustment for multiple comparisons (9 comparisons, *P* = 0.0045). gp120-specific IgG also showed high proportions of fucosylated and lower-bisecting glycans. Interestingly, the level of sialylation of gp120-specific IgG was higher than that in bulk IgG. This is in contrast to chronically HIV-infected adults, where gp120-specific antibodies were previously shown to have lower levels of sialylation than bulk IgG (21). The difference between p24- and gp120-specific bisecting glycosylation met the Bonferroni-adjusted significance threshold (9 comparisons, *P* = 0.0045).

10.1128/mSphere.00880-20.2 FIG S2 Fc glycosylation of antigen-specific IgG by disease phenotype. (A) Glycosylation patterns for p24-specific IgG stratified for HIV-infected progressor (Prog) (red) (*n* = 23) compared to nonprogressor (PNP) (blue) (*n* = 10) children. Median values are indicated by bar graphs, and interquartile ranges are indicated by error bars. Statistical comparisons between groups are based on Mann-Whitney U tests. (B) Same as in panel A but for gp120-specific IgG. Download FIG S2, PDF file, 0.1 MB. Copyright © 2020 Muenchhoff et al. 2020 Muenchhoff et al. This content is distributed under the terms of the Creative Commons Attribution 4.0 International license.

### PNPs demonstrate increased HIV-specific Fc-mediated IgG effector functions.

**FIG 4:**
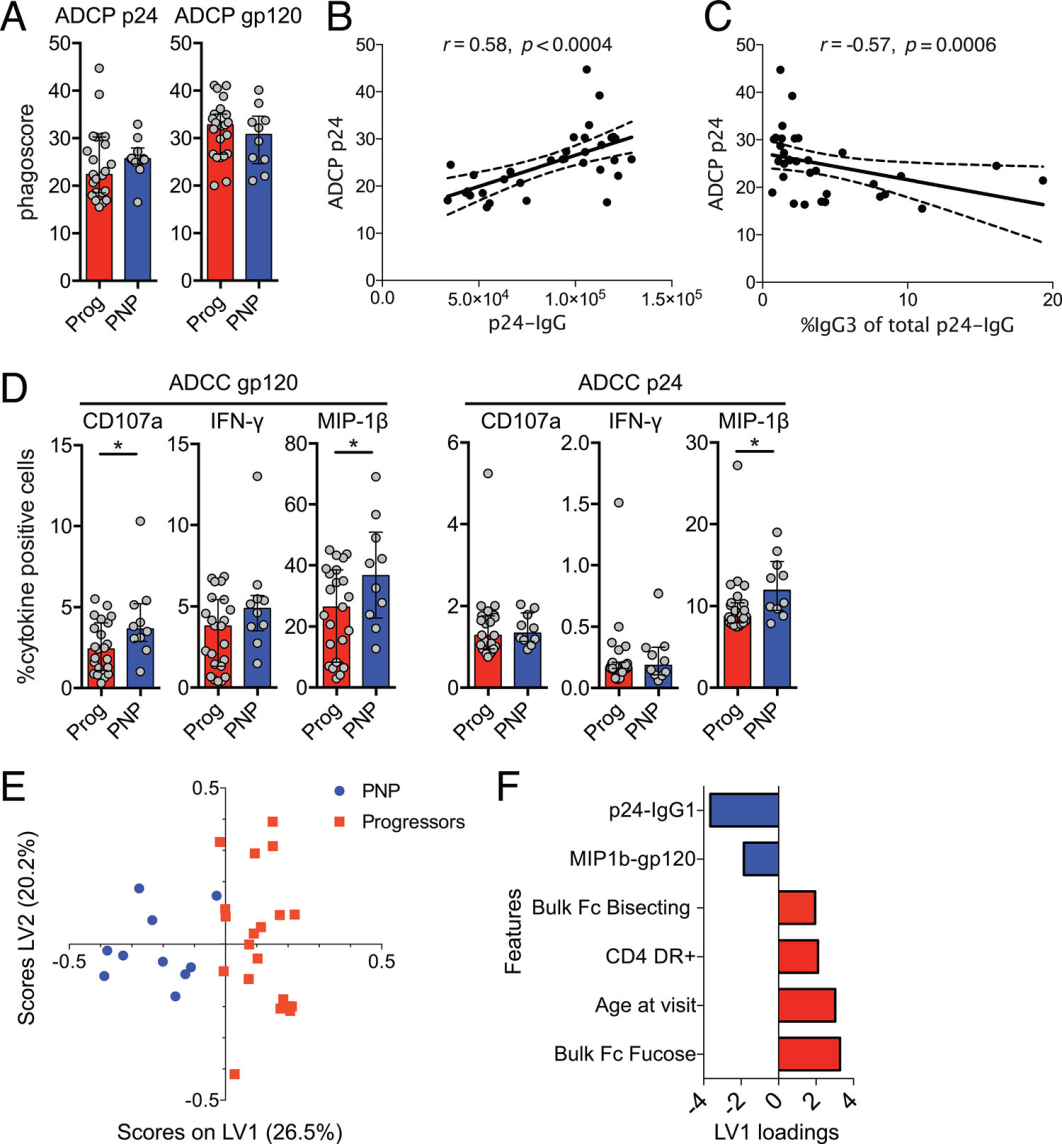
Fc-mediated effector functions are enhanced in nonprogressive pediatric HIV infection. (A) Antibody-dependent cellular phagocytosis represented as a phagoscore (percentage of positive cells times the mean fluorescence intensity [MFI]) for p24 (ADCP p24) and gp120 (ADCP gp120) antigens comparing progressor (red) and nonprogressor (blue) children. Median values are indicated by bar graphs, and interquartile ranges are indicated by error bars. (B and C) Spearman rank correlation between ADCP p24 phagoscores and p24 IgG titers (MFI) (B) and proportion of IgG3 of total p24-specific IgG (C) with trend lines and 95% confidence intervals (dotted lines). (D) Antibody-dependent cellular cytotoxicity (ADCC)-mediated NK-cell activation shown as frequencies of IFN-γ-, CD107a-, and MIP-1β-expressing NK cells using gp120 and p24 antigens. Medians and interquartile ranges are shown, and significance was tested using Mann-Whitney U tests (*, *P* < 0.05). (E) Immunological and clinical data were compiled and normalized by the Z-score. CD4^+^ cell counts were excluded from this analysis as cohort groups were stratified using this clinical variable. Feature selection by penalty-based least absolute shrinkage and section operator (LASSO) analysis was conducted and identified 6 of the original 49 features (see Table S2 in the supplemental material). PLSDA (partial least-squares discriminant analysis) scores plot where PNPs and progressors are represented for each child (dot), with a total *x* variance of 46.8% across the two dimensions (individual variance displayed on latent variable 1 [LV1] and LV2 axis labels), with 95% calibration, and with leave-one-out cross-validation success of 95%. (F) Corresponding loading bar plot showing the 6 selected features across LV1.

Beyond neutralizing activity, HIV-specific antibodies can mediate antibody-dependent cellular cytotoxicity (ADCC) and phagocytosis (ADCP) by binding to Fc receptors on effector cells such as NK cells and macrophages (18). To test if these antibodies could mediate immune effector functions and are associated with disease progression in pediatric HIV infection, we studied ADCP activity in an *in vitro* model, measuring THP-1 phagocytosis of p24 and gp120 antigen-coated beads opsonized with purified sample IgG. We observed marginally higher ADCP in PNPs for p24 antigen (median phagoscore, 22.3 versus 25.7; *P* = 0.31) but not gp120 antigen (Fig. 4A), likely due to the higher p24 IgG titers in PNPs and the correlation of p24 IgG levels with p24-specific ADCP (Fig. 4B). Interestingly, p24 antigen ADCP was inversely correlated with the IgG3 ratio of the total p24-specific IgG response (Fig. 4C).

ADCC responses were tested by NK-cell degranulation and intracellular cytokine staining after exposure to p24 and gp120 antigens incubated with purified IgG. PNPs showed higher ADCC activity, with statistically significant differences for gp120-specific CD107a and macrophage inflammatory protein 1β (MIP-1β) and p24-specific MIP-1β responses (Fig. 4D). These Fc-mediated cytokine functions were strongly correlated among each other; e.g., gp120-specific MIP-1β responses strongly correlated with gp120-specific CD107a responses (*r* = 0.93; *P* < 0.0001) (Table S1), indicating a higher capacity of HIV-specific IgGs in PNPs to mediate a broad range of NK-cell effector functions via Fc receptors.

10.1128/mSphere.00880-20.4 TABLE S1 Correlation of Fc-mediated NK-cell responses. Spearman’s rank correlations are shown for gp120-specific (top) and p24-specific (bottom) Fc-mediated NK-cell responses. Cytokine, chemokine, and degranulation marker responses are highly correlated as the reciprocal of the plasma dilution required to inhibit 50% of virus infection (ID_50_). Data are color coded as a heat map, with titers of >1,000 shown in red, those between 100 and 999 in orange, and those of <100 in light yellow. Titers of 1:40 indicate no neutralization (shown in blue). Murine leukemia virus (MuLV) was included as a negative control. Download Table S1, PDF file, 0.1 MB. Copyright © 2020 Muenchhoff et al. 2020 Muenchhoff et al. This content is distributed under the terms of the Creative Commons Attribution 4.0 International license.

10.1128/mSphere.00880-20.5 TABLE S2 List of parameters included in the LASSO and PLSDA models. The 49 parameters with available data for all participants that were used for the multivariate analysis are shown. Download Table S2, PDF file, 0.04 MB. Copyright © 2020 Muenchhoff et al. 2020 Muenchhoff et al. This content is distributed under the terms of the Creative Commons Attribution 4.0 International license.

### Unique immunological profiles distinguish PNP from progressor children.

In order to identify the minimum immunological features that best distinguish PNPs from progressors, we compiled immunological experimental and clinical data that were available for all subjects (total number of features, 49) (Table S2). We conducted feature selection by LASSO (least absolute shrinkage and selection operator) to select the minimal features, followed by PLSDA (partial least-squares discriminant analysis) to illustrate the features that best distinguish the two cohorts. Only 6 of the original 49 immunological and clinical features were required to separate PNPs (blue) and progressor children (red), with clear separation appearing to occur across the *x* axis (Fig. 4E). The right feature plot (Fig. 4F) spatially depicts the 6 selected features that distinguish the two cohorts. Consistent with previous analyses, p24-specific IgG1 and gp120-specific antibody-mediated NK-cell MIP-1β chemokine secretion were the two key features associated with PNP children, emphasizing the potentially important role of these antigen-specific humoral responses in disease nonprogression. Higher levels of MIP-1β responses have previously been associated with slower disease progression in adults, hypothesizing that this chemokine, as the natural ligand of CCR5, can block R5-tropic infection (26, 27). Because of the strong correlation between the different Fc-mediated NK-cell effector functions (Table S1 and Fig. S3), only one of the gp120-specific IgG-mediated responses, the MIP-1β response, was selected in the LASSO analysis, which is a feature selection model that often selects a single strong feature if several highly correlated predictors are present (28); thus, this can be regarded as being representative of increased Ab-mediated NK-cell activity, including other cytokine responses and NK-cell CD107a degranulation.

10.1128/mSphere.00880-20.3 FIG S3 Correlation matrix of immunological and clinical parameters. Spearman’s rank correlations are shown for a subset of 38 variables of all 33 progressor and nonprogressor children in this study. Positive correlations are indicated in blue, and inverse correlations are indicated in red. Darker color shades represent higher *r* values on the basis of Spearman’s rank correlation tests. *r* values are indicated at the bottom of the matrix, and *P* values are indicated by asterisks (*, *P* < 0.05; **, *P* < 0.01; ***, *P* < 0.001; ****, *P* < 0.0001). Clustering of variables is based on principal-component analysis using the R package corrplot. The same set of variables is used in the data model analysis (Fig. 4; see also Table S2 in the supplemental material), in addition to IgG subclass responses, which are not included here for clarity. Download FIG S3, PDF file, 0.1 MB. Copyright © 2020 Muenchhoff et al. 2020 Muenchhoff et al. This content is distributed under the terms of the Creative Commons Attribution 4.0 International license.

Not surprisingly, and consistent with our previous findings, increased CD4 T-cell activation (CD4 DR^+^) and age at visit, which reflect the duration of infection within children, were associated with progressive disease (2). Interestingly, specific signatures of IgG glycosylation, i.e., higher levels of fucosylated and bisecting glycans, that affect Fc receptor binding and hence IgG functionality were associated with disease progression.

### bnAb responses are correlated with circulating Tfh cells and gp120-specific IgG sialylation.

**FIG 5:**
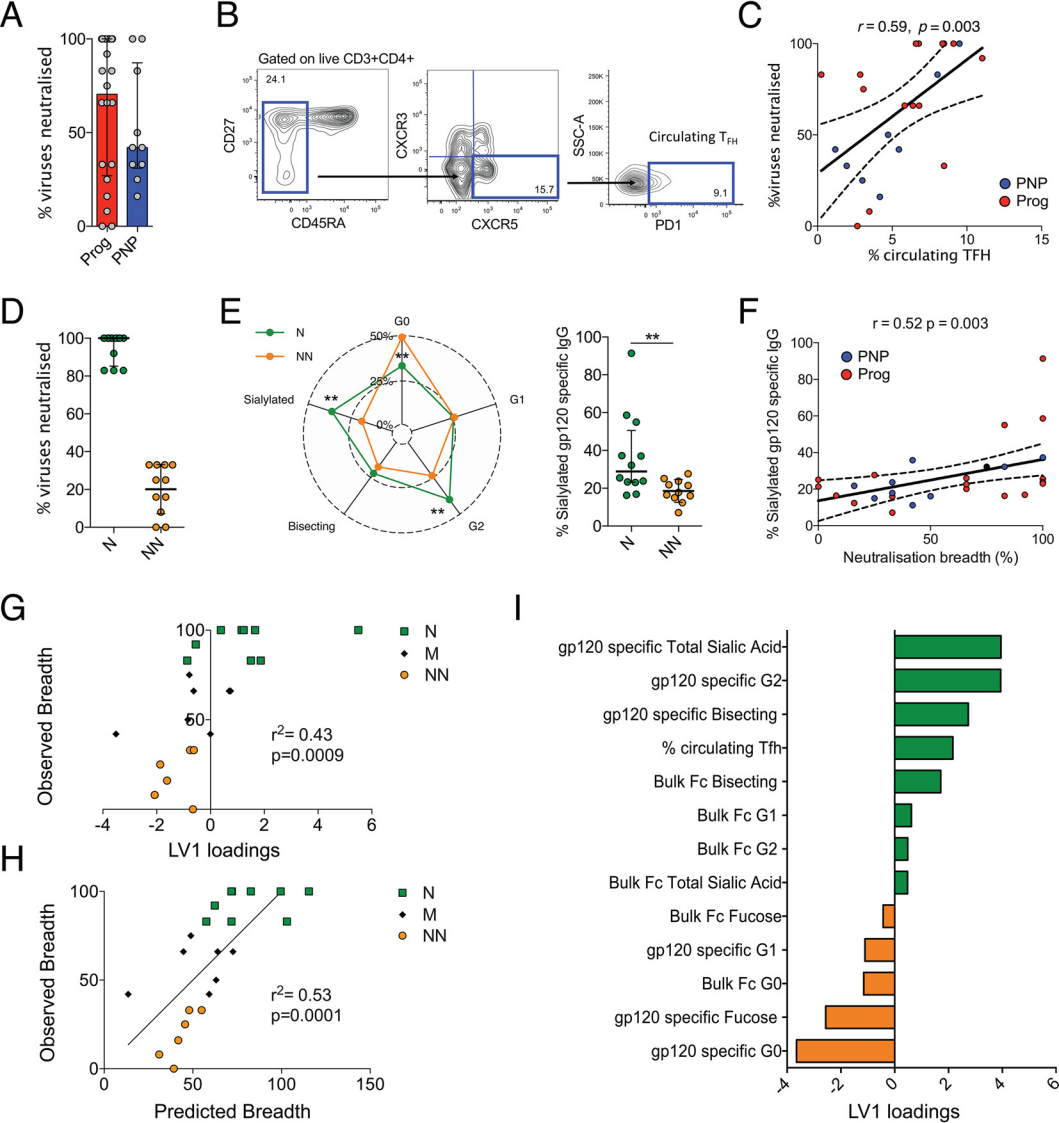
Broadly neutralizing antibody responses are associated with higher levels of circulating T follicular helper cells and gp120-specific Fc glycan sialylation. (A) Neutralization breadth and geometric mean neutralization titers against a standardized global 12-virus panel (29) presented as medians (bars) and interquartile ranges (error bars) for progressor children (red) and nonprogressor children (blue). (B) Gating strategy for circulating Tfh cells defined as PD-1^+^ CXCR5^+^ CXCR3^−^ CD45RA^−^ CD3^+^ CD4^+^ T cells. SSC-A, side scatter area. (C) Spearman rank correlation between neutralization breadth and circulating Tfh frequencies with trend lines and 95% confidence intervals (dotted lines). (D) Percentages of viruses neutralized by neutralizers (N) (green) and nonneutralizers (NN) (orange). (E) gp120-specific Fc glycosylation profiles of neutralizers (green) and nonneutralizers (orange). Significance was tested using Mann-Whitney U tests (**, *P* < 0.01). (F) Spearman rank correlation between neutralization breadth and Fc glycan sialylation with trend lines and 95% confidence intervals (dotted lines). (G) Partial least-squares regression (PLSR) conducted on 17 Fc glycan measurements and Tfh features to determine the relationship with neutralization breadth, with a total *x* variance of 25.5% across latent variable 1 (LV1), a root mean square error calibration (RMSEC) value of 24.12, and a root mean square error cross-validation (RMSECV) value of 30.33. Neutralizer, medium neutralizer (M), and nonneutralizer breadths (*y* axis) were plotted against LV1. loadings. (H) Calculated predicted breadths using two dimensions of PLSR analysis (LV1 and LV2 scores) resulting in a combined *x* variance of 44.4%, an RMSEC value of 21.8, and an RMESC value of 32.5. (I) Corresponding features used in PLSR analysis plotted against LV1 *x*.

HIV-infected children develop broadly neutralizing antibodies faster and at higher frequencies than adults (2, 4). We measured serum neutralization activity against a consensus global panel of 12 viruses (29) (Table S3) and detected potent bnAb responses in most children, with a trend toward higher neutralization breadths and titers in progressors than in PNPs, consistent with our previous study (2) (Fig. 5A). Circulating PD-1^+^ CXCR3^−^ CXCR5^+^ memory Tfh cells have been previously associated with HIV neutralization breadth by us (8) and others (7, 30). Confirming this relationship, we observed a correlation between circulating Tfh cells (defined as PD-1^+^ CXCR5^+^ CXCR3^−^ CD45RA^−^ CD4 T cells) (see gating in Fig. 5B) and neutralization breadth in our cohort (*r* = 0.59; *P* = 0.002) (Fig. 5C).

10.1128/mSphere.00880-20.6 TABLE S3 Breadth of plasma neutralizing activity. Plasma samples from 20 progressor and 9 nonprogressor children (highlighted in green) were tested against a consensus global panel of 16 heterologous viruses (29) using the TZM-bl neutralization assay. Neutralization titers are shown. Download Table S3, PDF file, 0.6 MB. Copyright © 2020 Muenchhoff et al. 2020 Muenchhoff et al. This content is distributed under the terms of the Creative Commons Attribution 4.0 International license.

We then identified neutralizers (N), children with the capacity to neutralize >80% of viruses (from the above-mentioned global panel [29]), and nonneutralizers (NN), children that neutralized <35% of viruses (Fig. 5D). The N group includes 4 PNP and 7 progressor children, and the NN group consists of 3 PNP and 9 progressor children. Recent studies suggested that IgG Fc glycosylation can affect affinity maturation of antibody responses by increased complement deposition and higher affinity for Fc receptors, resulting in an enhanced delivery of immune complexes to the germinal center for antigen presentation (31, 32). Significantly higher levels of gp120-specific Fc sialylation and G2 (*P* = 0.0056 and *P* = 0.01, respectively) were observed in pediatric neutralizers (Fig. 5E). Indeed, gp120-specific Fc sialylation was strongly correlated with neutralization breadth (*r* = 0.52; *P* = 0.003) (Fig. 5F). Neutralization breadth was also positively correlated with gp120-specific G2 (*r* = 0.47; *P* = 0.009) and negatively correlated with G0 (*r* = −0.48; *P* = 0.007) Fc glycan abundances, likely because galactosylation is a prerequisite for the addition of sialic acid residues.

In order to compare the associations of multiple IgG Fc glycosylation structures and Tfh cells (*n* = 13 variables) with neutralization breadth, we conducted partial least-squares regression (PLSR) and observed that latent variable 1 (LV1) was able to moderately predict breadth (Fig. 5G) (*r*^2^ = 0.43; *P* = 0.0009; *x* variance, 25.5%; *y* variance, 43.2%). The inclusion of the first two dimensions of the PLSR analysis (LV1 and LV2) improved the predicted breadth (Fig. 5H) (*r*^2^ = 0.53; *P* = 0.0001; combined *x* variance, 44.4%; combined *y* variance, 53.4%). Not surprisingly, sialylated G2 glycans and circulating Tfh cells were features that correlated with greater neutralizing breadth (Fig. 5I).

## DISCUSSION

In the quest for an HIV vaccine, research focus has recently shifted toward the pediatric population due to both the necessity to establish protection before sexual debut and the observation that vertically HIV-infected children mount broad and potent neutralizing antibody responses by as early as 1 year of age (2, 4). The immune system in early life constitutes functional Tfh responses and a general Th2 bias compared to adults and is therefore geared toward the effective induction of potent antibody responses, making children a promising target population for an HIV vaccine (1, 8). Nonneutralizing antibodies have been associated with protection against HIV infection, possibly due to Fc-regulated effector functions, including ADCC, ADCP, and the activation of the complement system (10, 33). These biological activities of an antibody are regulated by glycosylation of the Fc part of IgG, modulating the binding affinity for different Fc receptors (18). However, little is known so far about Fc glycosylation and its immunoregulatory effects in children with HIV infection. In the present study, we consider both neutralizing and nonneutralizing antibody responses together with IgG glycosylation patterns and correlate them with T-cell parameters to gain a better understanding of the pediatric immune landscape and how it could be harnessed for vaccine strategies.

Our finding that PNPs have significantly increased p24 IgG/IgG1 titers is consistent with at least 15 previous studies demonstrating slower disease progression in patients with higher IgG levels against HIV-1 Gag proteins (reviewed in reference 34). However, the causal relationship of this observation remains unclear. Despite previous reports about p24 antigen being expressed on the surface of infected cells (35) and viral inhibition and ADCC mediated by p24-specific monoclonal antibodies in *in vitro* infection models (36), it remains largely unknown to what extent p24-specific antibodies could mediate antiviral effects *in vivo*. Studies in chronically HIV-infected adults showed increased opsonophagocytic IgG responses against p24 in HIV controllers with low levels of viremia, but it remains challenging to distinguish between cause and effect in these observational studies (37). In fact, it has been speculated that lower p24-specific IgG levels in progressive disease are due to the loss of p24-specific CD4 T-cell help (38). However, in contrast to this hypothesis, we observed that PNPs had overall lower levels of HIV-specific CD4 T-cell responses and circulating bulk Tfh cells than progressors, consistent with the general PNP phenotype of low immune activation. We observed a greater contribution of the IgG3 subclass to the p24-specific IgG response in progressor children. IgG3 antibodies effectively mediate proinflammatory effector functions and usually display a shorter half-life than other IgG subclasses, possibly to limit the potential of an excessive inflammatory response (39). Their increased frequency in progressing children and association with CD4 counts might reflect the increased immune activation observed in this group. A recent study in HIV-infected adults demonstrated that p24 IgG1 titers were a correlate of viral control independent of antigen-specific CD4 or CD8 T-cell responses (40), thus suggesting additional, as-yet-unclear roles for p24-specific IgG as a surrogate marker of delayed disease progression, which deserves further consideration.

Even though there is growing evidence that antibody glycosylation is an actively controlled process, the underlying immunological mechanisms are poorly understood (reviewed in reference 18). While physiological conditions, including age, sex, smoking, and body mass index (41), have been associated with different bulk glycosylation levels, there is literature linking agalactosylated and asialylated carbohydrate forms of antigen-specific IgGs with chronic inflammation in both autoimmune diseases (20, 23) and chronic HIV infection (21). In the present study, in contrast to progressor children, PNPs showed no significant perturbations in bulk IgG glycosylation compared to HIV-uninfected children. Interestingly, this is in stark contrast to the typical phenotype of adult HIV long-term nonprogressors, the so-called “elite controllers,” who also display overall less immune activation than progressors but with pronounced inflammatory IgG signatures (21). These data confirm and highlight that low levels of systemic immune activation are a cardinal feature of nonprogressive pediatric HIV infection despite persistent viremia.

Here, compared to bulk IgG, gp120-specific IgGs showed high fractions of agalactosylated (G0) structures at the expense of G1, while no differences were observed for p24, indicating differential regulation of glycan structures against different antigens of the same pathogen. This suggests that these differences are not related purely to systemic inflammation, which would presumably impact all antigen specificities equally. In addition, the level of sialylation of gp120-specific IgG was higher than that of bulk IgG, which is in contrast to studies of chronically HIV-infected adults where gp120-specific antibodies showed lower levels of sialylation than did bulk IgG (21). This observation is particularly interesting in light of a recent study showing an increase in B-cell affinity maturation and the development of bnAbs against HIV after the administration of immune complexes with sialylated antigen-specific antibodies (31). This is thought to be related to enhanced immune complex deposition in germinal centers. Consistent with this, we observed a strong correlation between gp120-specific Fc sialylation and neutralization breadth in our cohort, suggesting that this a viable mechanism for enhanced affinity maturation in infants. Since galactosylation is a prerequisite for sialylation, G2 glycoforms and sialylation levels are interdependent variables, and the associations with neutralization breadth apply to both parameters. Mechanistically, sialylation of the IgG Fc glycan results in a “closed” conformational change with a lower affinity for activating type I but a higher binding affinity for type II Fc receptors modulating anti-inflammatory properties and driving B-cell affinity maturation via increased interaction with DC-Sign in germinal centers (32, 42). Of note, IgG galactosylation, which is a prerequisite for sialylation, is known to strongly vary by age, with steadily increasing nongalactosylated and decreasing digalactosylated glycans (43). Experience from vaccine studies suggests that while there are a variety of factors that can impact baseline antibody glycosylation, the specificity of the changes after vaccination speaks for an antigen-specific regulation of glycosylation at the time of immune priming (44, 45). The precise mechanisms by which this is controlled remain unclear and may be context specific, but there is evidence that B-cell stimulation by IL-21 can enhance galactosylation and sialylation (46). In our previous report, we showed that HIV-specific Tfh cells in secondary lymphoid tissue of HIV-infected children responded mainly with IL-21, whereas the Tfh response in adults was dominated by IFN-γ (8), suggesting a potential mechanism for increased sialylation in this group. Importantly, these differences were not observable in circulation, where IL-21-producing Tfh cells were extremely rare and inconsistently detected.

The potential role of nonneutralizing antibody responses in HIV infection and prevention was put into the limelight when immune correlate studies from the RV144 trial identified an association between Env-gp120-specific IgG antibodies capable of mediating ADCC *in vitro* and a lower risk of infection (11, 47). Although not as effective as neutralizing antibodies, elegant *in vivo* studies confirmed that nonneutralizing antibodies can mediate protection against and modify the course of HIV infection in an Fc-dependent manner in mice (48). In breastfed infants from HIV-infected mothers, higher levels of maternal HIV-specific antibodies in breast milk that were able to mediate ADCC were associated with a reduced risk of transmission (16). Passively transferred maternal IgG mediating ADCC in vertically infected children was associated with better clinical outcomes and higher survival rates during infancy and up to 2 years of age (17). In our study of children well beyond infancy, we also find that *de novo* IgG responses that mediate higher levels of NK-cell effector functions are associated with better clinical outcomes and slower disease progression. Of note, gp120-specific IgG titers were not increased in PNPs, suggesting that the higher ADCC functionality was mediated by qualitative rather than quantitative differences in gp120-specific IgG in PNPs compared to progressors. In this context, PNPs showed lower levels of fucosylated bulk IgG than progressors. Afucosylation directly increases ADCC activity by enhancing binding to Fc gamma receptor IIIa and is therefore used to enhance the therapeutic effects of monoclonal antibodies (49). Hence, the increased ADCC in PNPs might be related to the lower proportions of fucosylated bulk IgG observed in this group. Alternatively, the lower degrees of activation and maintenance of CD4 T-cell help in nonprogressor children were contributing to the development or maintenance of functional antibodies in this group, rather than these antibodies preventing disease progression.

These data suggest exciting potential roles of nonneutralizing HIV-specific antibodies in mediating HIV nonprogression and generating broadly neutralizing antibodies. These features may be exploited in rational vaccine design or attempts to achieve HIV cure or remission. However, a major limitation of this study is the small sample size of our PNP cohort. Results should be confirmed in larger cohort studies that could additionally also represent a broader age spectrum from infancy to early adulthood. Due to the observational character of this study, we are not able to distinguish between cause and effect but report solely on correlations. The use of recombinant antigens in their nonnative configuration for antibody capture and in some of the assay systems used in this study does not fully mimic the epitope configuration *in vivo* and may lead to biased results. Future studies should investigate ADCC and ADCP function using *in vitro* infection models, ideally with autologous infected cells. We did not further examine the potential mechanisms that may underlie the differential regulation of IgG glycosylation and function, but this could be an interesting avenue for future projects to comprehensively map the immune landscape in early life and how it could be harnessed for vaccine strategies. Taken together, our data suggest that early-life immunization might favor the induction of bnAb responses by increased Tfh activity and Fc sialylation in response to a prophylactic vaccine.

## MATERIALS AND METHODS

### Study design.

The goals of this study were to identify unique immunological properties of HIV-infected children with broadly neutralizing antibody responses and a nonprogressive disease phenotype. We used systems immunology, computational analysis, enzyme-linked immunosorbent assays (ELISAs), glycan analysis, Luminex, TZM-bl neutralization assays, flow cytometry, and Fc-mediated phagocytosis and cytokine release assays to study samples from HIV-uninfected and -infected children. Throughout the initial Fc profiling, the samples were blinded and unblinded for analysis.

### Study subjects.

Vertically HIV-infected children and adolescents were recruited at the Ithembalabantu Clinic in Umlazi, Durban, South Africa. Three groups of vertically HIV-infected children above 5 years of age were recruited based on the following criteria: (i) pediatric nonprogressor (PNP) children (*n* = 10), who were ART naive, had a CD4 count of >750 cells/mm^3^, and were asymptomatic; (ii) pediatric progressors (Prog) (*n* = 23), who were ART naive and had a CD4 count of <500 cells/mm^3^; and (iii) ART-treated children (ART) (*n* = 13), who were currently receiving ART and had undetectable viral loads. Additionally, 10 HIV-negative siblings were recruited. Clinical characteristics of the study subjects are summarized in Table 1. Study participants were selected from a previously described larger cohort (2) based on sample availability. Viral load and CD4 count measurements were performed as previously described (2). Informed written consent was obtained from the caregivers, and additionally, assent to participate in the study was given by all children above 6 years of age. Studies were approved by the Biomedical Research Ethics Committee, University of KwaZulu-Natal, Durban, and the Research Ethics Committee, University of Oxford.

### Sample preparation.

After plasma separation, peripheral blood mononuclear cells (PBMCs) were isolated from EDTA-blood by Ficoll-Hypaque density gradient centrifugation and used directly or cryopreserved in 90% fetal calf serum (FCS) plus 10% dimethyl sulfoxide (DMSO) in liquid nitrogen. Cryopreserved PBMCs were thawed and rested in medium (RPMI 1640 [Sigma-Aldrich] plus 10% FCS and 50 U penicillin-streptomycin) before use.

### IgG purification.

IgG was isolated from plasma samples using Melon Gel IgG purification resin (Thermo Fisher) according to the manufacturer’s instructions, a method that avoids low-pH aggregation of IgG (50).

### Antigen-specific IgG subclass profiles.

To determine the relative antigen-specific HIV-1 clade C IgG subclass (IgG1, IgG2, IgG3, and IgG4) levels in plasma, we modified a customized subclass multiplex binding assay as previously described (51). Briefly, carboxylated microsphere beads (Luminex) were coupled to recombinant proteins of interest: p24 (clade C) (GenBank accession no. AY463234), gp120 (clade C/Du151), gp140 (clade C/Du151), and gp41 (ΔTM [transmembrane]) (clade C/ZA.1197MB) (all from Immune Technology). Purified bulk IgG for each sample was added to 5 wells of a 96-well plate and incubated overnight at 4°C. The supernatant was washed away, and individual IgG isotype detection reagent, bulk IgG, IgG1, IgG2, IgG3, or IgG4 conjugated with phycoerythrin (PE) (Southern Biotech), was then added individually to each of the 5 wells containing bound sample antibody. The 96-well plate was incubated with shaking for 2 h, washed, and read on a Bio-Plex 200 system Luminex instrument.

### Antigen stimulation and intracellular cytokine staining.

PBMCs were adjusted to 1 million cells/stimulation and stimulated using peptide pools (18-mers overlapping by 10 amino acids) covering the HIV-1 clade C consensus Gag, Pol, Env, Nef, regulatory (Reg) (Tat and Rev), or accessory (ACC) (Vpr, Vpu, and Vif) proteins (2 μg/ml per peptide) as described previously (52). PBMCs were stimulated at 37°C in the presence of the costimulatory antibodies anti-CD28 and anti-CD49d (BD Bioscience) at 1 μg/ml. After 1 h of incubation, brefeldin A (Sigma-Aldrich) and monensin were added at 10 μg/ml. Following incubation overnight (12 to 16 h), intracellular cytokine staining was performed according to standard methods (53). Briefly, cells were washed and stained in the dark for 20 min with a cell surface antibody cocktail and live/dead stain (near infrared [IR]; Invitrogen). Subsequently, cells were fixed and permeabilized using BD Cytofix/Cytoperm buffer and stained for intracellular cytokines with antibodies in BD Perm/Wash buffer (BD Biosciences). Reagents and panels are shown in Table S4 in the supplemental material. Samples were acquired on a BD LSRII instrument, and data were analyzed using FlowJo v10.0.7 (TreeStar). Frequencies of cytokine-positive cells were background subtracted using an unstimulated control without the addition of antigen for each patient.

10.1128/mSphere.00880-20.7 TABLE S4 Flow cytometry panels and reagents. The fluorochrome-conjugated antibodies used in this study are indicated with the clone number and supplier. Download Table S4, PDF file, 0.04 MB. Copyright © 2020 Muenchhoff et al. 2020 Muenchhoff et al. This content is distributed under the terms of the Creative Commons Attribution 4.0 International license.

### Phenotypic flow cytometry.

PBMCs were stained with fluorescent monoclonal antibodies against a panel of markers previously associated with Tfh cells (8) and a panel of markers for immune activation/memory differentiation (Table S4). Rainbow beads were run in every experiment to ensure interexperimental consistency. Flow cytometry acquisition was performed on a BD LSRFortessa instrument within 5 h of staining and analyzed using FlowJo versions 9.9.5 and 10.0.7.

### IgG glycosylation.

Antigen-specific IgGs were purified for glycan analysis as previously described (54). Bulk or antigen-specific Fc glycosylation was conducted as previously reported, using recombinant p24 and gp120 antigens based on HIV-1 subtype C consensus sequences (clade C/Du151) (25). Briefly, purified IgG or antigen-specific IgG was denatured and treated with peptide-*N*-glycosidase (PNGase) (New England BioLabs [NEB]). Proteins were precipitated in ethanol, and the glycan-containing supernatants were dried using CentriVap and labeled with APTS (3-aminopropyltriethoxysilane; Thermo Fisher). Samples were run on an ABI 3130XLI DNA sequencer.

### Antibody-dependent cellular cytotoxicity.

A modified ELISA-based assay for the detection of CD107a as a surrogate marker of NK-cell-mediated degranulation and cytolysis was performed as previously described (40). Briefly, a 96-well ELISA plate was coated overnight at 4°C with recombinant protein. Purified IgG was added to each well, and the plate was incubated at 37°C for 2 h. HIV-negative plasma samples or medium alone was used as a negative control, while HIVIG (pooled HIV immunoglobulin G) (NIH AIDS Reagents Program) was used as a positive control. A total of 5 × 10^4^ NK cells enriched via negative selection from healthy blood donors (RosetteSep; Stemcell Technologies) were added to each well in the presence of brefeldin A (BioLegend), Golgi stop, and anti-CD107a-PE-Cy5 (BD Biosciences). Plates were incubated for 5 h at 37°C with 5% CO_2_. Cells were then stained with anti-CD3-AlexaFluor700, anti-CD56-PE-Cy7, and anti-CD16-allophycocyanin (APC)-Cy7 (BD); fixed with Perm A; permeabilized using Perm B (Invitrogen); and stained with anti-IFN-γ–APC and anti-MIP-1β–PE (BD). The cells were fixed with a 2% paraformaldehyde solution and analyzed by flow cytometry.

### Antibody-dependent cellular phagocytosis.

A THP-1 antibody-dependent cellular phagocytosis (ADCP) assay was performed as previously described (55). Briefly, recombinant HIV proteins were conjugated onto 1-μm fluorescent neutravidin beads (Invitrogen). Excess antigen was removed by washing pelleted beads, which were then incubated with purified IgG antibody samples (100 μg/ml) for 2 h at 37°C. Following opsonization, THP-1 cells were added to the bead-antibody mix and incubated overnight, followed by fixation and analysis of bead uptake by flow cytometry.

### Neutralization assays.

The ability of plasma from HIV-infected children to neutralize HIV-1 was measured against an established “global” panel of 12 viruses (29) as previously described (2).

### Statistical analysis.

Statistical analysis was undertaken using GraphPad Prism software version 6.0. For exploration of bivariate associations, Spearman’s rank correlation test was used. *P* values for comparisons between groups were calculated using Mann-Whitney tests. All *P* values are two sided, and a *P* value of less than 0.05 was considered significant. In scatterplots, median values and interquartile ranges (IQRs) are indicated. Due to the observatory nature of this study, *P* values were initially not corrected for multiple comparisons. For the main analyses shown in Fig. 1 to 3, *P* values were adjusted for multiple comparisons using the Bonferroni method as reported in Results.

In addition to these traditional statistical comparisons, we performed multivariate model analyses with unbiased system serology approaches using the least absolute shrinkage and selection operator (LASSO) and partial least-squares discriminant analysis (PLSDA). Key minimal features that contributed to differences between cohorts (PNPs and progressors) were identified using the LASSO penalized regression feature selection method (56). Cross-validation was performed iteratively (repeated 10,000 times; 10-fold cross-validation) to find the optimal value of the regularized parameters. PLSDA was conducted to discriminate differences between two cohorts (PNPs and progressors) in multivariate space. Partial least-squares regression (PLSR) was conducted to determine the multivariate relationship between immune features and continuous variables (breadth). Prior to analysis, all data were normalized with mean centering and variance scaling. The analysis was conducted using Matlab with the statistics and machine learning toolbox (MathWorks) and PLS_Toolbox (Eigenvector). A correlation matrix was plotted to visualize Spearman’s correlations using the corrplot package in R with grouping of variables on the basis of principal-component analysis.
