# Supplementary figures and images for: Distinct Immunoglobulin Fc Glycosylation Patterns Are Associated with Disease Nonprogression and Broadly Neutralizing Antibody Responses in Children with HIV Infection
Source: mSphere. 2020 Dec 23;5(6):e00880-20. doi: 10.1128/mSphere.00880-20 (PMC7763548; doi:10.1128/mSphere.00880-20)

A

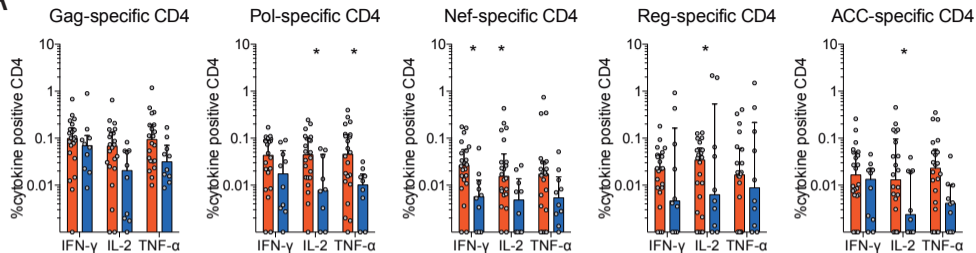

B

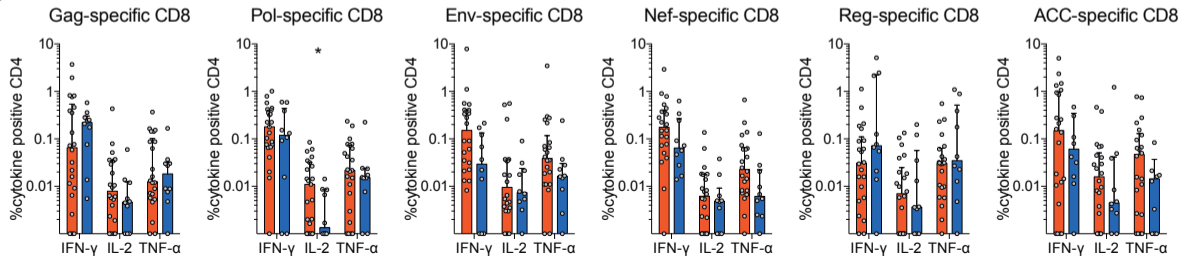

Supplement: FIG S1 [file mSphere.00880-20-sf001.pdf]

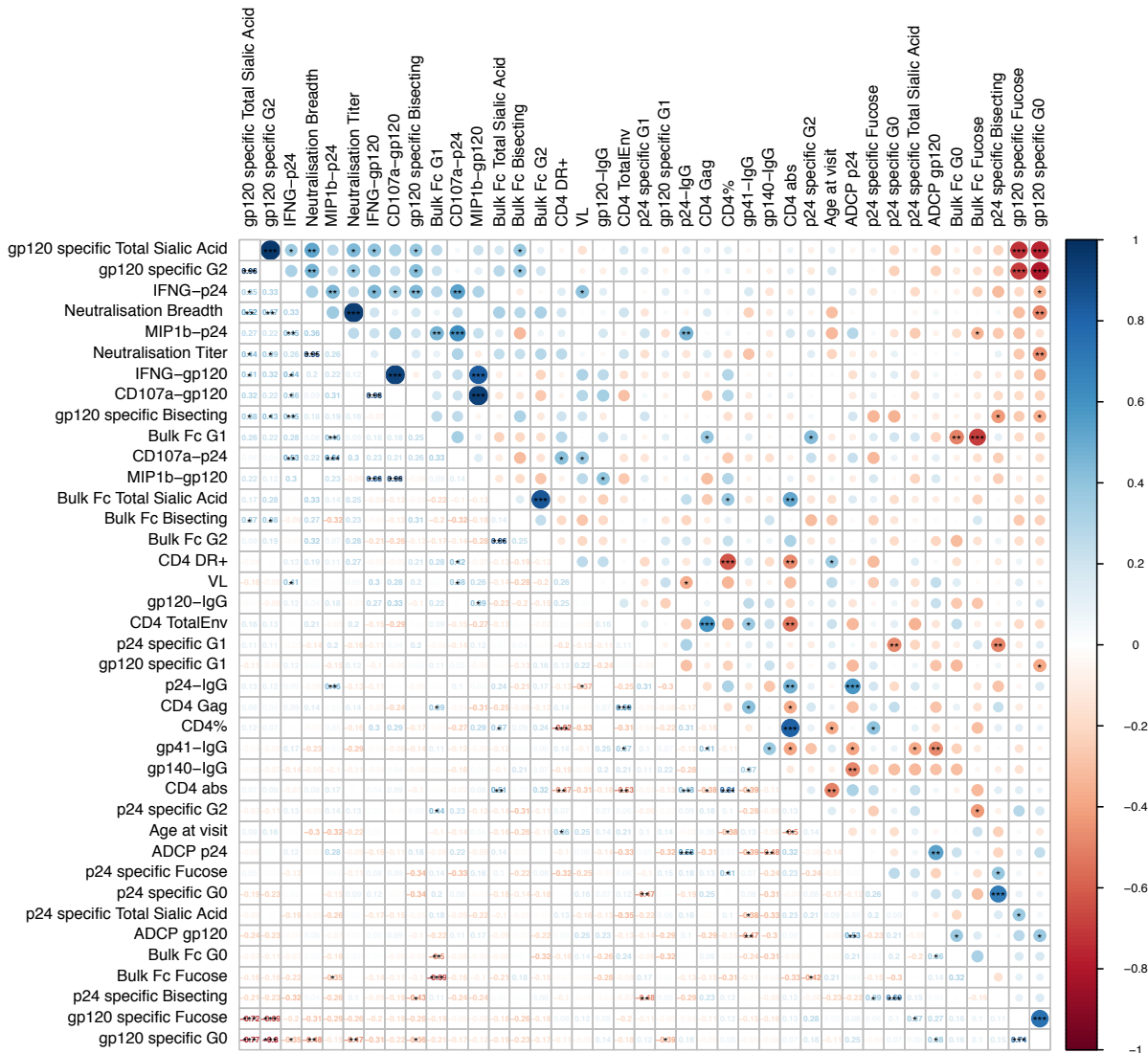

Supplement: FIG S3 [file mSphere.00880-20-sf003.pdf]
