# Supplementary material for: Distinct Immunoglobulin Fc Glycosylation Patterns Are Associated with Disease Nonprogression and Broadly Neutralizing Antibody Responses in Children with HIV Infection
Source: mSphere. 2020 Dec 23;5(6):e00880-20. doi: 10.1128/mSphere.00880-20 (PMC7763548; doi:10.1128/mSphere.00880-20)
Supplement: FIG S2 [file mSphere.00880-20-sf002.pdf]

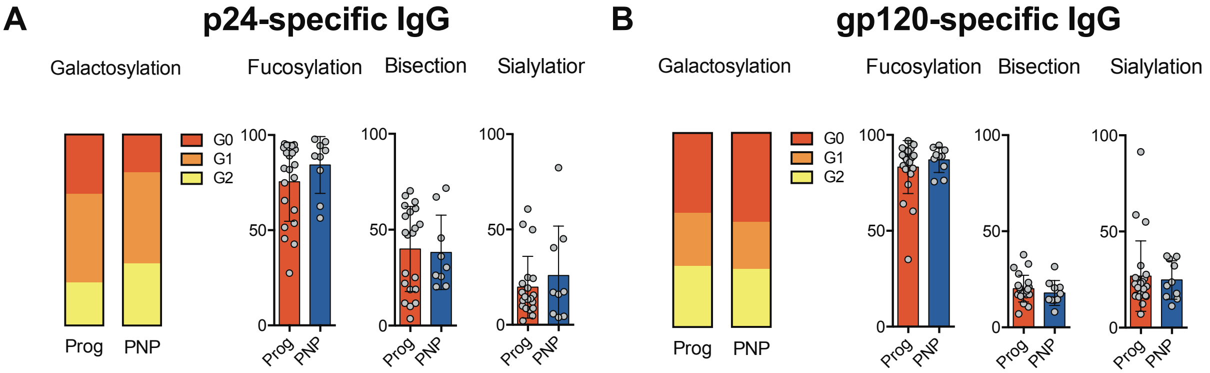

A
p24-specific IgG
Galactosylation
Fucosylation
Bisection
Sialylatior
G0
G1
G2
100
50
0
Prog
PNP
B
gp120-specific IgG
Galactosylation
Fucosylation
Bisection
Sialylation
