# Supplementary material for: Distinct Immunoglobulin Fc Glycosylation Patterns Are Associated with Disease Nonprogression and Broadly Neutralizing Antibody Responses in Children with HIV Infection
Source: mSphere. 2020 Dec 23;5(6):e00880-20. doi: 10.1128/mSphere.00880-20 (PMC7763548; doi:10.1128/mSphere.00880-20)
Supplement: TABLE S1 [file mSphere.00880-20-st001.pdf]

gp120

	MIP-1beta	CD107a	IFN-gamma
**MIP-1beta**		r=0.93, p<0.0001	r=0.94, p<0.0001
**CD107a**	r=0.93, p<0.0001		r=0.83, p<0.0001
**IFN-gamma**	r=0.94, p<0.0001	r=0.83, p<0.0001	

p24

	MIP-1beta	CD107a	IFN-gamma
**MIP-1beta**		r=0.53, p=0.0015	r=0.61, p=0.0002
**CD107a**	r=0.53, p=0.0015		r=0.45, p=0.009
**IFN-gamma**	r=0.61, p=0.0002	r=0.45, p=0.009	
