# Supplementary material for: Distinct Immunoglobulin Fc Glycosylation Patterns Are Associated with Disease Nonprogression and Broadly Neutralizing Antibody Responses in Children with HIV Infection
Source: mSphere. 2020 Dec 23;5(6):e00880-20. doi: 10.1128/mSphere.00880-20 (PMC7763548; doi:10.1128/mSphere.00880-20)
Supplement: TABLE S2 [file mSphere.00880-20-st002.pdf]

Number	Feature
1	Age at visit
2	Gender
3	Viral Load
4	ADCP gp120
5	ADCP p24
6	CD107a-gp120
7	IFNG-gp120
8	MIP1b-gp120
9	CD107a-p24
10	IFNG-p24
11	MIP1b-p24
12	gp120-IgG
13	gp41-IgG
14	p24-IgG
15	gp140-IgG
16	gp120-IgG1
17	gp41-IgG1
18	p24-IgG1
19	gp140-IgG1
20	gp120-IgG2
21	gp41-IgG2
22	p24-IgG2
23	gp140-IgG2
24	gp120-IgG3
25	gp41-IgG3
26	p24-IgG3
27	gp140-IgG3
28	gp120-IgG4
29	gp41-IgG4
30	p24-IgG4
31	gp140-IgG4
32	gp120 specific G0
33	gp120 specific G1 (with & without Sialylation)
34	gp120 specific G2 (with & without Sialylation)
35	gp120 specific Fucose (with & without Sialylation)
36	gp120 specific Bisecting (with & without Sialylation)
37	gp120 specific Total Sialic Acid
38	Bulk Fc G0
39	Bulk Fc G1 (with & without Sialylation)
40	Bulk Fc G2 (with & without Sialylation)
41	Bulk Fc Fucose (with & without Sialylation)
42	Bulk Fc Bisecting (with & without Sialylation)
43	Bulk Fc Total Sialic Acid
44	CD4 DR+
45	CD4 Total (IforILorT) Gag
46	CD4 Total (IforILorT) Env
47	Nab Breadth
48	Nab Titer (geo. Mean)
49	Nab Titer (normal average)
