# Supplementary material for: Distinct Immunoglobulin Fc Glycosylation Patterns Are Associated with Disease Nonprogression and Broadly Neutralizing Antibody Responses in Children with HIV Infection
Source: mSphere. 2020 Dec 23;5(6):e00880-20. doi: 10.1128/mSphere.00880-20 (PMC7763548; doi:10.1128/mSphere.00880-20)
Supplement: TABLE S4 [file mSphere.00880-20-st004.pdf]

Panel intracellular cytokine staining

Antibody	Flurochrome	clone	supplier
CD3	BV605	OKT3	BioLegend
CD4	BV510	L200	BD
CD8	BV421	RPA-T8	BD
TNF-alpha	PE-Cy7	MAb11	BD
IL-2	FITC	5344.111	BD
IFN-gamma	AlexaFluor700	B27	BD
Live/dead	Near-IR	N/A	Invitrogen

Panel memory differentiation/activation

Antibody	Flurochrome	clone	supplier
CD3	BV605	OKT3	BioLegend
CD4	BV510	L200	BD
CD8	BV421	RPA-T8	BD
CD38	PE-Cy7	HIT2	BD
CCR7	PE	#150503	R&D systems
HLA-DR	FITC	L243	BD
CD45RA	AlexaFluor700	HI100	BioLegend
Live/dead	Near-IR	N/A	Invitrogen

Panel T-follicular helper cells

Antibody	Flurochrome	clone	supplier
CD69	BUV 395	FN50	BD
CCR6	BUV 496	11A9	BD
PD1	BV 421	EH12.1	BD
CD8	V500	RPA-T8	BD
CCR4	BV 605	L291H4	biolegend
CD45RA	BV 650	HI100	BD
CD25	BV 711	BC96	biolegend
CD3	BV 785	OKT3	biolegend
CXCR5	AlexaFluor 488	RF8B2	BD
CCR7	PerCp-Cy5-5	G043H7	biolegend
CD127	PE-Cy5	R34.34	Beckmann Coulter
CD27	PE-Cy7	M-T271	biolegend
CXCR3	PE-CF594	1C6/CXCR3	BD
CD40L	PE	24-31	biolegend
ICOS	Alexa Fluor 647	C398.4A	biolegend
CD4	AlexaFluor 700	RPA-T4	BD

Panel ADCC

Antibody	Flurochrome	clone	supplier
CD3	AlexaFluor 700	UCHT1	BD
CD16	APC-Cy7	3G8	BD
CD56	PE-Cy7	B159	BD
IFN-gamma	APC	B27	BD
MIP1-beta	PE	D21-1351	BD
CD107a	PE-Cy5	H4A3	BD
